# Mesenchymal Stem/Stromal Cell Therapy Is More Cost-Effective Than Fecal Diversion for Treatment of Perianal Crohn’s Disease Fistulas

**DOI:** 10.3389/fimmu.2022.859954

**Published:** 2022-06-17

**Authors:** Sheeva Johnson, Jeffrey S. Hoch, Wissam J. Halabi, Jeffrey Ko, Jan Nolta, Maneesh Dave

**Affiliations:** ^1^Division of Gastroenterology and Hepatology, Department of Internal Medicine, University of California (UC) Davis Medical Center, University of California Davis School of Medicine, Sacramento, CA, United States; ^2^Division of Health Policy and Management, Department of Public Health Sciences, University of California (UC) Davis, Davis, CA, United States; ^3^Division of Colorectal Surgery, Department of Surgery, University of California (UC) Davis Medical Center, University of California Davis School of Medicine, Sacramento, CA, United States; ^4^Department of Internal Medicine, Institute for Regenerative Cures, University of California (UC) Davis, Sacramento, CA, United States

**Keywords:** Crohn’s disease, perianal fistula, fecal diversion, mesenchymal stem cells, cost effectiveness

## Abstract

Crohn’s disease (CD) is an inflammatory bowel disease with increasing incidence and prevalence worldwide. Perianal fistulas are seen in up to 26% of CD patients and are often refractory to medical therapy. Current treatments for CD perianal fistulas (pCD) include antibiotics, biologics, and for refractory cases, fecal diversion (FD) with ileostomy or colostomy. Mesenchymal stem/stromal cell therapy (MSCs) is a new modality that have shown efficacy in treating pCD. MSCs locally injected into pCD can lead to healing, and a phase III clinical trial (ADMIRE-CD) showed 66% clinical response, leading to approval of MSCs (Alofisel, Takeda) in the European Union. It is unclear if MSCs would be more cost-effective than the current standard of FD. We therefore developed a decision tree model to determine the cost-effectiveness of MSCs compared to FD for pCD. Our study showed that both autologous and allogeneic MSCs are more cost-effective than FD in an academic medical center and even in a worst-case scenario with 100% chance of all complications for MSCs treatment and 0% chance of complications for FD, both allogeneic and autologous MSCs are still cost saving compared to FD.

## Introduction

Crohn’s disease (CD) is an inflammatory bowel disease (IBD) with increasing incidence and prevalence worldwide (1). Perianal fistulas are seen in up to 26% of CD patients and are often refractory to medical therapy (2). Current treatments for CD perianal fistulas (pCD) include antibiotics, biologics, and for refractory cases, fecal diversion (FD) with ileostomy or colostomy (2). In cases where temporary FD is performed, restoration of bowel continuity is performed in less than half the cases, and 41.6% ultimately require proctocolectomy (3).

Mesenchymal stem/stromal cell therapy (MSCs) is a new therapeutic modality that has shown efficacy in treating refractory pCD (4). MSCs have been demonstrated to exert their clinical effects through a variety of anti-inflammatory and tissue regenerative mechanisms (4). MSCs locally injected into perianal fistulas can lead to healing (4), with a meta-analysis demonstrating healing rates of 64% (4). A subsequent phase III clinical trial (ADMIRE-CD) showed 66% clinical response at 1 year, leading to approval of MSCs (Alofisel, Takeda) for pCD in the European Union (5).

The costs of care for patients with IBD have been rising over time, with one driver of these costs being novel therapeutics (6). There is thus an imperative for the development of cost-effective therapies and strategies for patients with IBD. As a novel therapeutic modality, it is unclear if MSCs would be more cost-effective than the current standard of FD for pCD, which has similar clinical response (5). Thus, the objective of our study was to determine the cost-effectiveness of MSCs compared to FD for pCD.

## Methods

**Figure 1:**
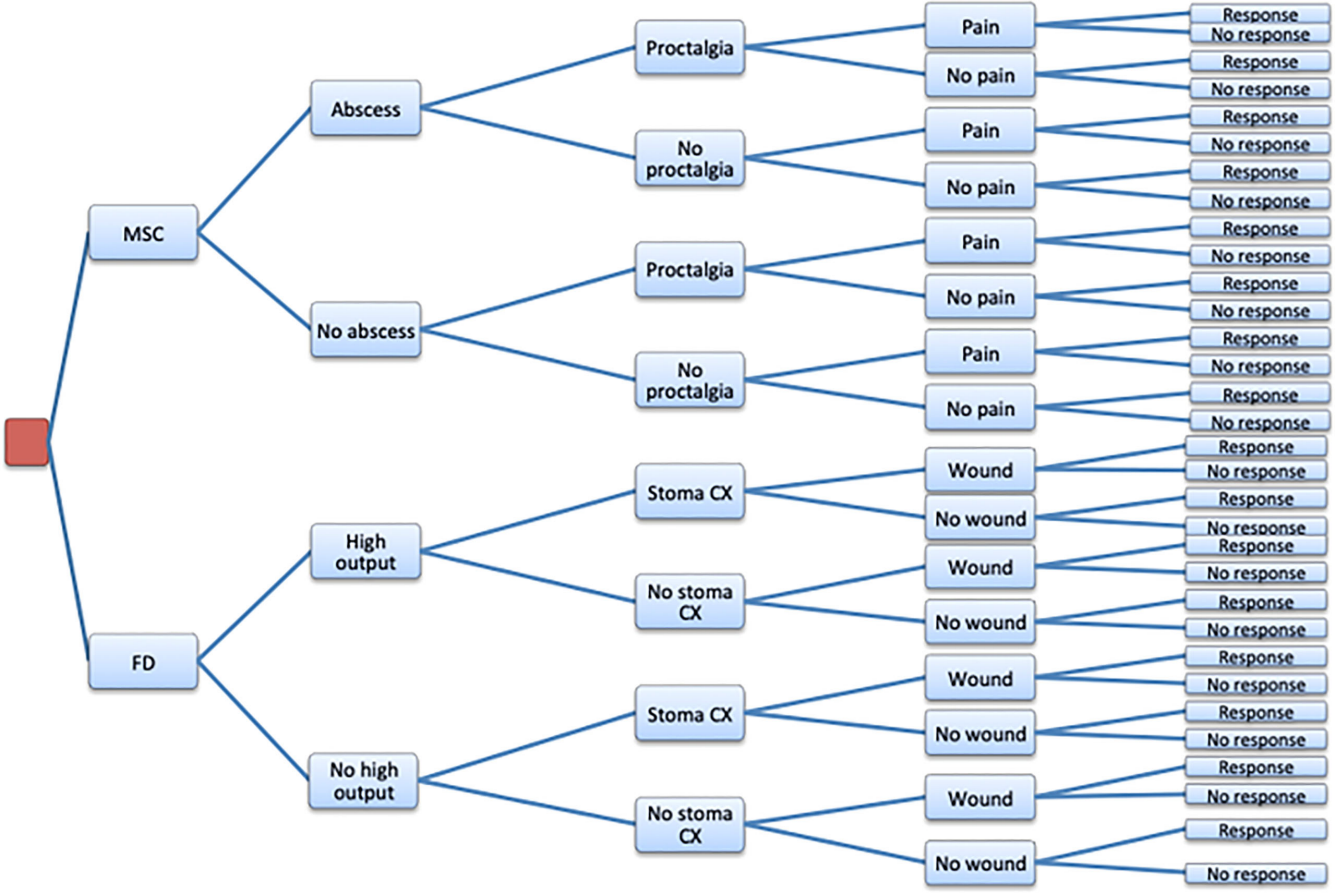
Decision Tree Model. MSC, Mesenchymal stem cell theraphy; FD, Fecal Diversion; Abscess, Anal abscess; High output, Dehydration or renal failure from high ostomy output; Proctalgia, Protalgia; Pain, Procedural pain; Stoma CX, Stoma related compilations: parastomal hernia, retraction hernia, retraction, prolapse, obstruction; Wound, Peristomal skin related complications; Response, Clinical response.

A decision tree model was developed to compare the cost-effectiveness of MSCs vs. FD in the treatment of pCD over a 52-week period (**Figure 1**). Both allogeneic and autologous MSCs were separately compared to FD. A hypothetical cohort of patients with refractory pCD were studied receiving MSCs or FD. Economic modeling has been used successfully by us to investigate cost-effectiveness in many conditions including gastrointestinal diseases (7).

The probabilities of complications for FD were determined from a review of previously published studies (see **Supplemental References**) and included dehydration, stoma-related complications, and wound care. Frequency of dehydration was 15% (lower range frequency 11%, upper range frequency 20%). Stoma-related complications, which included parastomal hernia, retraction, prolapse, and obstruction, occurred with 26.5% frequency (lower range frequency 17%, upper range frequency 40%). Wound care and skin breakdown occurred with 31% frequency (lower range frequency 20%, higher range frequency 36%). The probabilities of the most common treatment-related adverse effects for MSCs were obtained from the ADMIRE-CD clinical trial and included anal abscess (13%), proctalgia (5%), and procedural pain (1%) (5). The items to be included for cost estimation for FD, treatment with MSCs, and managing their respective complications including office visits, hospital admission, imaging studies, exam under anesthesia (EUA), ostomy supplies, and medications were decided in consultation with a colorectal surgeon (WJH) (see **Supplementary Table 1**). Total cost of stoma-related complications was calculated from a combination of costs of conservative management (96% of stoma-related complications) and revision surgery (4% of stoma-related complications). Costs are described in US dollars. The costs for treatment and complications arising from treatment were determined using 2017 Centers for Medicare & Medicaid Services (CMS) databases using national average (see **Supplementary Table 2**). Costs for the production and administration of allogeneic and autologous MSCs were based on Good Manufacturing Practice (GMP) grade cells for clinical trial use from the University of California, Davis Institute for Regenerative Cures (see **Supplementary Table 2**).

Effectiveness was defined as clinical response for each treatment. Clinical response for FD was defined as general clinical improvement, estimated at 63.8% as determined from a systematic review and meta-analysis (3). Clinical response for MSCs was defined as closure of at least 50% of all treated external openings that were draining at baseline, estimated at 66% in ADMIRE-CD (5). This definition of clinical response for MSCs was chosen instead of clinical remission (closure of 100% of fistulous openings), as there are no comparable published outcomes for FD where closure of fistulous openings was evaluated as a primary outcome. Furthermore, the 66% response is nearly identical to the 64% healing rate we previously computed in a meta-analysis (4). A model where the more stringent definition of clinical remission for effectiveness of MSCs, as determined in ADMIRE-CD (5), was also used to compare to clinical response in FD.

The cost-effectiveness of MSCs compared to FD was evaluated comparing expected costs and expected effectiveness. The incremental cost-effectiveness ratio (ICER) was calculated to describe the economic attractiveness of MSCs compared to FD. Sensitivity and scenario analyses were also performed as logic checks and to test the sensitivity of our findings.

## Results

In the base case analysis for allogeneic MSCs, one administration of allogeneic MSCs had an expected total cost of $13,536, and FD an expected cost of $17,642 (**Table 1**). The difference in expected cost was -$4,106 (MSCs cost less than FD). The cost savings is primarily driven by the difference in treatment cost of $3,603. The difference in costs for expected complications is $503 less for MSCs than FD. The expected clinical response is 2.2% greater for MSCs than FD. Even using a more stringent endpoint of clinical remission (59.2%) compared to FD’s clinical response (63.8%), the extra cost of FD’s improved clinical response is substantial with an ICER of $91,035.

**Table 1:** Base Case Cost-Effectiveness.

Treatment option	Expected Effect	Expected Total Cost	Expected Treatment Cost	Expected Complication Cost
**FD**	0.638	$17,642	$16,868	$774
**MSC allogeneic**	0.66	$13,536	$13,265	$271
**MSC autologous**	0.66	$7,536	$7,265	$271
**Compared to FD**				
**MSC allogeneic**	0.022	$4,106 savings	$3,603 savings	$503 savings
**MSC autologous**	0.022	$10,106 savings	$9,603 savings	$503 savings

In conclusion, our study demonstrates that MSCs are more cost effective than FD for refractory pCD in an academic medical center.

In the base case analysis for autologous MSCs, one administration of autologous MSCs had an expected total cost of $7,536, while the expected cost for FD remained unchanged at $17,642 (**Table 1**). Thus, the additional expected cost of autologous MSCs vs. FD is -$10,106. The difference in expected complications cost is $503 less for MSCs than FD. Using the stringent endpoint of clinical remission for autologous MSCs compared to clinical response in FD, the economic attractiveness of FD is questionable with a substantial ICER of $221,470.

In a worst-case scenario analysis with 100% chance of all complications for MSCs and 0% chance of complications for FD, both allogeneic and autologous MSCs remain cost saving compared to FD (FD is $95 more than allogeneic and $367,850 more than autologous MSCs). Additional one-way and two-way sensitivity analyses confirmed the cost-effectiveness of MSCs.

## Discussion

Our study is one of the first to directly compare the costs of MSCs to FD for treatment of pCD. Allogeneic and autologous MSCs were less costly than FD when factoring the cost of the therapy and expected complications; moreover, autologous MSCs appear to be more cost-effective than allogeneic MSCs in our study. Costs were modeled from the payer perspective using Medicare costs, where reimbursement is lower than commercial insurance. Thus, costs of FD and cost of complications from FD are underestimated, meaning the estimated cost savings from MSCs are likely greater than what is described in our present study. Considering that the complications of MSCs per ADMIRE-CD (anal abscess, proctalgia, and procedure-related pain) (5) are less burdensome than the typical complications of FD (dehydration, wound care, stoma-related complications) with regards to hospitalization, resources, and surgical management, this likely represents a more marked benefit for the patient from both the cost and healthcare utilization standpoints.

Clinical effectiveness of FD in this study, defined as a general clinical improvement as determined by meta-analysis of FD for pCD (3), overestimates actual fistula healing and closure of fistulous openings for FD, thus likely biasing our results towards FD. Many prior studies on FD have described symptomatic improvement but do not report actual objective fistula healing rate as determined by no fistula drainage despite gentle finger compression, exam under anesthesia, MRI pelvis, or endoscopic ultrasound. Our recent study evaluating fistula healing after FD as a primary endpoint showed fistula healing of 40% and is similar to two recent multicenter studies which reported between 36.8-42.2% fistula healing after FD, which is much lower than the summary estimate of 63.8% used in our study (8–10). At the same time, the MSC fistula healing rates are obtained from a robust phase III clinical trial (ADMIRE-CD) (5), and is similar to those reported in multiple studies including recent systematic reviews, meta-analysis and compassionate use programs where patients with perianal fistulas refractory to surgical intervention were treated with MSCs (4, 11, 12). Therefore, our analysis that was biased towards FD still supports the cost effectiveness of MSCs compared to FD for pCD over a 52-week period.

A limitation of our study is that it utilizes economic modeling instead of conducting a clinical trial to investigate MSCs and FD in pCD; however economic modeling is commonly used to examine cost-effectiveness in a wide variety of settings where trials are infeasible or unpractical, including in gastrointestinal diseases (7). In this setting, no head-to-head comparative studies evaluating MSCs vs. FD are available and should be a focus of future studies. We did not compare IBD medications in our model as patients in both groups have refractory pCD (FD and MSC) and are likely on immunosuppressive IBD medications for treatment of luminal CD. The costs of MSCs were based on GMP grade cells for clinical trial use in academic medical center, and thus may not be reflective of all GMP-certified facilities but are still estimated to be much are lower than commercial sources. A similar paradigm can be seen in other cell therapy products like CAR T-cell therapies, where in-house GMP facilities at academic centers have much lower costs compared to their commercial counterparts (13).

In conclusion, our study demonstrates that MSCs are more cost-effective than FD for refractory pCD in an academic medical center.

## Data Availability Statement

The original contributions presented in the study are included in the article/**Supplementary Material**. Further inquiries can be directed to the corresponding author.

## Author Contributions

SJ (Methodology: supporting, Data curation: lead; Formal analysis: supporting; Writing- original draft: lead), JH (Methodology: lead; Formal analysis: lead; Writing- Review and editing: supporting; Supervision: supporting), WJH (Methodology: supporting; Writing- Review and editing: supporting), JK (Data curation: supporting; Writing- Review and editing: supporting), JN (Methodology: supporting; Writing- Review and editing: supporting), MD (Conceptualization: lead; Methodology: supporting, Formal analysis: supporting; Writing- Review and editing: lead; Supervision: lead). All authors contributed to the article and approved the submitted version.

## Funding

MD is supported by K08DK110421.

## Conflict of Interest

The authors declare that the research was conducted in the absence of any commercial or financial relationships that could be construed as a potential conflict of interest.

## Publisher’s Note

All claims expressed in this article are solely those of the authors and do not necessarily represent those of their affiliated organizations, or those of the publisher, the editors and the reviewers. Any product that may be evaluated in this article, or claim that may be made by its manufacturer, is not guaranteed or endorsed by the publisher.
