# Supplementary material for: Mesenchymal Stem/Stromal Cell Therapy Is More Cost-Effective Than Fecal Diversion for Treatment of Perianal Crohn’s Disease Fistulas
Source: Front Immunol. 2022 Jun 17;13:859954. doi: 10.3389/fimmu.2022.859954 (PMC9248358; doi:10.3389/fimmu.2022.859954)
Supplement: Supplementary file 1 [file Table_1.docx]

Supplementary Table 1. Determination of costs

| Procedure/Complication | Items from which costs estimated |
| --- | --- |
| MSC Allogeneic | Pre-operative visit, post-operative visit, EUA* x 2, MSC cost |
| MSC Autologous | Pre-operative visit, post-operative visit, EUA* x 2, MSC cost |
| Fecal Diversion | Pre-operative visit, post-operative visit, EUA* x 1, surgical cost including hospital admission, 52 weeks of ostomy supplies |
| Wound care/skin breakdown | Office visit x 2 |
| Dehydration/Renal failure | One hospital admission |
| Stoma complications-  Revision surgery (4%) | Surgical cost including hospital admission, office visit x 2, CT abdomen/pelvis |
| Stoma complications-  Conservative management (96%) | Office visit x 2, CT abdomen/pelvis, ostomy belt |
| Anal abscess/fistula | Image-guided drainage, office visit x 1, CT abdomen/pelvis |
| Proctalgia | Office visit x 1, EUA* x 1 |
| Procedural pain | Acetaminophen 325 mg tab x 30 tabs |

*Exam under anesthesia

Supplementary Table 2. Costs of treatments and complications

|  | Base case  costs | Minimum  cost | Maximum  costs | References |
| --- | --- | --- | --- | --- |
| MSC Allogeneic | $13,265 | $12,789 | $14,138 | IRC** |
| MSC Autologous | $7,265 | $6,789 | $8,138 | IRC** |
| Fecal Diversion | $16,868 | $13,272 | $24,529 | CMS*** |
| Wound care/skin breakdown | $155 | $143 | $208 | CMS*** |
| Dehydration/Renal failure | $3,477 | $2,674 | $6,460 | CMS*** |
| Stoma complications –  conservative management * | $540 | $528 | $594 | CMS*** |
| Stoma complications – revision * | $6,362 | $4,804 | $10,696 | CMS*** |
| Anal abscess | $1,430 | $1,254 | $1,683 | CMS*** |
| Proctalgia | $1,705 | $1,700 | $17,27 | CMS*** |
| Procedural pain | $3 | $1 | $7 | ^@^ |

**Estimated from University of California, Davis Institute for Regenerative Cures (IRC)

***Centers for Medicare and Medicaid Services database (CMS) (https://data.cms.gov)

^@^https://www.goodrx.com/Tylenol

**Supplemental References**

1. Paquette IM, Solan P, Rafferty JF, Ferguson MA, Davis BR. Readmission for dehydration or renal failure after ileostomy creation. *Dis Colon Rectum.* 2013;56(8):974-979.

2. Hayden DM, Pinzon MC, Francescatti AB, et al. Hospital readmission for fluid and electrolyte abnormalities following ileostomy construction: preventable or unpredictable? *J Gastrointest Surg.* 2013;17(2):298-303.

3. Fish DR, Mancuso CA, Garcia-Aguilar JE, et al. Readmission After Ileostomy Creation: Retrospective Review of a Common and Significant Event. *Ann Surg.* 2017;265(2):379-387.

4. Caricato M, Ausania F, Ripetti V, Bartolozzi F, Campoli G, Coppola R. Retrospective analysis of long-term defunctioning stoma complications after colorectal surgery. *Colorectal Dis.* 2007;9(6):559-561.

5. Arumugam PJ, Bevan L, Macdonald L, et al. A prospective audit of stomas--analysis of risk factors and complications and their management. *Colorectal Dis.* 2003;5(1):49-52.

6. Marti-Gallostra M, Myrelid P, Mortensen N, Keshav S, Travis SP, George B. The role of a defunctioning stoma for colonic and perianal Crohn's disease in the biological era. *Scand J Gastroenterol.* 2017;52(3):251-256.

7. Robertson I, Leung E, Hughes D, et al. Prospective analysis of stoma-related complications. *Colorectal Dis.* 2005;7(3):279-285.

8. Park JJ, Del Pino A, Orsay CP, et al. Stoma complications: the Cook County Hospital experience. *Dis Colon Rectum.* 1999;42(12):1575-1580.
